# The algicidal mechanism of prodigiosin from *Hahella* sp. KA22 against *Microcystis aeruginosa*

**DOI:** 10.1038/s41598-017-08132-5

**Published:** 2017-08-10

**Authors:** Ke Yang, Qiuliang Chen, Danyang Zhang, Huajun Zhang, Xueqian Lei, Zhangran Chen, Yi Li, Yaling Hong, Xiaohong Ma, Wei Zheng, Yun Tian, Tianling Zheng, Hong Xu

**Affiliations:** 1 State Key Laboratory of Cellular Stress Biology, and School of Life Sciences, Xiamen University, Xiamen, Fujian 361102 P. R. China; 2 Key Laboratory of the Ministry of Education for Coastal and Wetland Ecosystems, College of the Environment and Ecology, Xiamen University, Xiamen, Fujian 361102 P. R. China

## Abstract

In recent years, *Microcystis aeruginosa* blooms have occurred throughout the world, causing huge economic losses and destroying aquatic ecosystems. It is necessary to develop effective and ecofriendly methods to control *M. aeruginosa* blooms. Here, we report a high algicidal activity of prodigiosin (PG) against *M. aeruginosa* as well as the algicidal mechanism. PG showed high algicidal activity against *M. aeruginosa*, with a 50% lethal dose (LD_50_) of 5.87 μg/mL in 72 h. A combination of methods, including propidium iodide and Annexin V-fluorescein staining assays and light and electron microscopy indicated the existence of two modes of cell death with features similar to those in eukaryotic programmed cell death: necrotic-like and apoptotic-like. Biochemical and physiological analyses showed that PG generates reactive oxygen species (ROS), which induce lipid peroxidation, damage the membrane system and destroy the function of the photosystem. A proteomics analysis revealed that many proteins were differentially expressed in response to PG stress and that most of these proteins were involved in important metabolic processes, which may trigger necrotic-like or apoptotic-like cell death. The present study sheds light on the multiple toxicity mechanisms of PG on *M. aeruginosa* and its potential for controlling the occurrence of *M. aeruginosa* blooms in lakes.

## Introduction

Due to increasing human activities, water eutrophication has become more serious throughout the world in recent decades. Water eutrophication can promote the proliferation of cyanobacteria, which eventually causes cyanobacterial blooms^1, 2^. Cyanobacterial blooms caused by toxic cyanobacteria, such as *Microcystis*, *Anabaena*, and *Cylindrospermopsis* ^3, 4^, frequently occur in fresh water lakes worldwide and have serious impacts on aquaculture industries and the drinking water supply^5^. *Microcystis aeruginosa*, a dominant species of bloom-forming cyanobacteria, can produce and release microcystins, which can accumulate in aquatic animals including molluscs, shrimp, and fish and then be transmitted through the food chain to humans^6, 7^. Microcystins can cause serious damage to the liver and ultimately lead to disease or death in humans^8^.

To prevent and eliminate cyanobacterial blooms of *M. aeruginosa*, many measures, such as physical (e.g. clay^9, 10^) and chemical methods (e.g. copper sulphate, hydrogen peroxide and potassium permanganate^11–13^), have been implemented. However, these methods are not only costly, but may also lead to secondary pollution of the aquatic environment. Therefore, the development of economical and ecofriendly approaches to control cyanobacterial blooms has important theoretical and practical significance. In recent years, studies on the biological control of cyanobacterial blooms have received widespread attention, especially in regard to microorganisms with algicidal activity^14, 15^. Many algicidal bacteria, such as *Bacillus* ^16, 17^, *Pseudoalteromonas* ^18^, *Pseudomonas* ^19, 20^, *Vibrio* ^21^, *Acinetobacter* ^22^ and *Alcaligenes* ^23^, have been reported to have the potential to control harmful algal blooms. Darveau and Lynch reported that *Serratia marcescens* could inhibit the growth of *Anabaena* by producing prodigiosin (PG)^24^. Since then, several studies have demonstrated the high algicidal activity of PG against *Cochlodinium polykrikoides* ^25^, *M. aeruginosa* ^26^ and *Phaeocystis globosa* ^27^. Prodigiosins (PGs) are a family of red pigments characterized by a common pyrrolyldipyrrolylmethene skeleton. For the past several decades, PGs have been extensively studied for their potential as antibiotics and antitumour drugs, owing to their broad range of cytotoxic activities^28, 29^. Although PG has shown high algicidal activity and the potential to control harmful algal blooms, the knowledge about its algicidal mechanism is still limited.

To investigate the algicidal mechanism of PG against *M. aeruginosa*, we extracted PG from cultures of *Hahella* sp. KA22. We treated *M. aeruginosa* with different concentrations of PG to investigate the effect of PG on cellular membrane permeability, DNA degradation, photosynthetic performance and antioxidant response of *M. aeruginosa* during cell death. Furthermore, to explore the molecular mechanism of PG-induced *M. aeruginosa* cell death, we employed the HPLC-MS-based TMT labelling technique to quantify the global proteome in *M. aeruginosa* and comparatively analysed the differentially expressed proteins at 12 h, 24 h, 48 h and 72 h after PG treatment of algal cultures.

## Results

### Algicidal activity and the alga-lytic process

**Figure 1:**
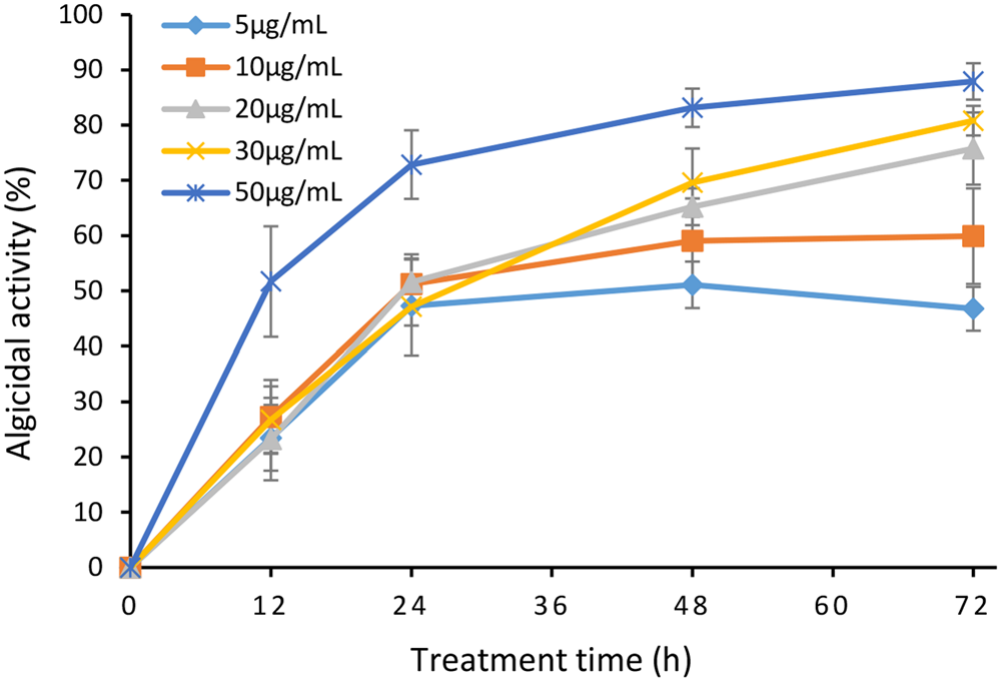
Algicidal activity of PG against *M. aeruginosa* at concentrations of 5, 10, 20, 30 and 50 μg/mL. The algicidal activity was detected at 12, 24, 48 and 72 h. All data are means ± SD (n = 3).

After being treated with various concentrations of PG for 12 h, more than 20% of the algal cells had been lysed. The algicidal effect increased with increasing concentrations of PG. At 50 μg/mL, 12 h of treatment resulted in the lysis of 52% of the algal cells (Fig. 1), and the algicidal activity of PG increased as treatment time was prolonged. Cells treated with concentrations of 5 and 10 μg/mL of PG exhibited 47% and 60% cell lysis respectively, within 72 h. The groups treated with higher concentrations (20, 30 and 50 μg/mL) displayed 76%, 81% and 88% algal cell lysis, respectively, at 72 h. PG showed high algicidal activity against *M. aeruginosa* with an LD_50_ of 5.87 μg/mL in 72 h. Based on the algicidal effects and the dose of PG, 30 μg/mL of PG demonstrated high algicidal activity and was chosen as the suitable concentration for further study.

**Figure 2:**
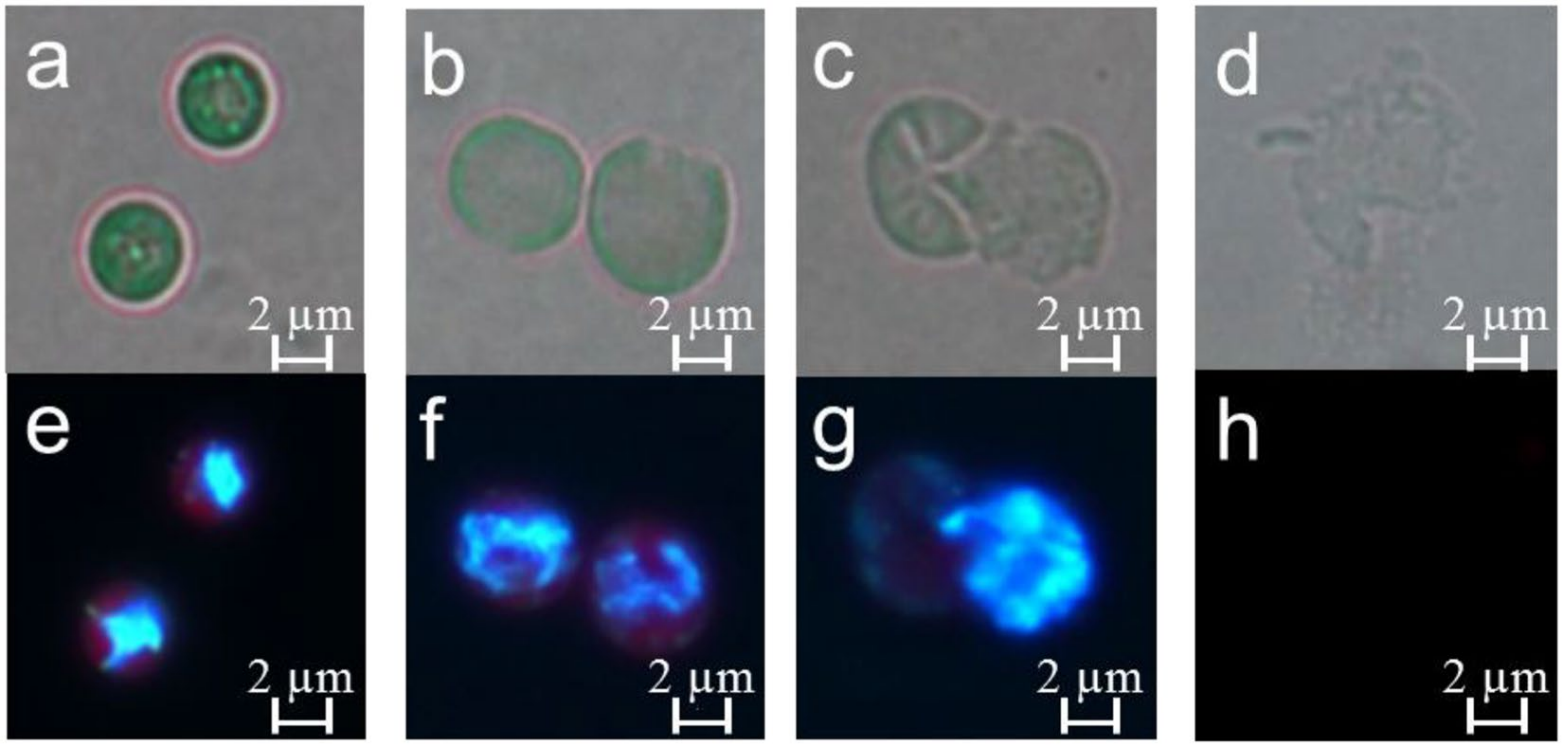
The effects of PG on cell morphology and nucleoids of *M. aeruginosa*. Algal cells were harvested by centrifugation after being exposed to PG for 0 (**a**,**e**), 24 (**b**,**f**), 48 (**c**,**g**) and 72 h (**d**,**h**) and were stained with DAPI (5 µg/mL) for 5 min in the dark at room temperature, and observed by fluorescence microscopy.

The alga-lysing process was observed under a microscope (Fig. 2). The lysis of the algal cells began with the loss of cell membrane integrity followed by penetration of PG into the cells, which caused most of the cells to swell (Fig. 2b). Subsequently, the cells burst and only cell debris remained (Fig. 2c,d). In the meantime, the nucleoids of the algal cells diffused to the entire cell upon the penetration of PG and were at last released from the cells (Fig. 2e,f,g).

### Cell membrane integrity analysis

**Figure 3:**
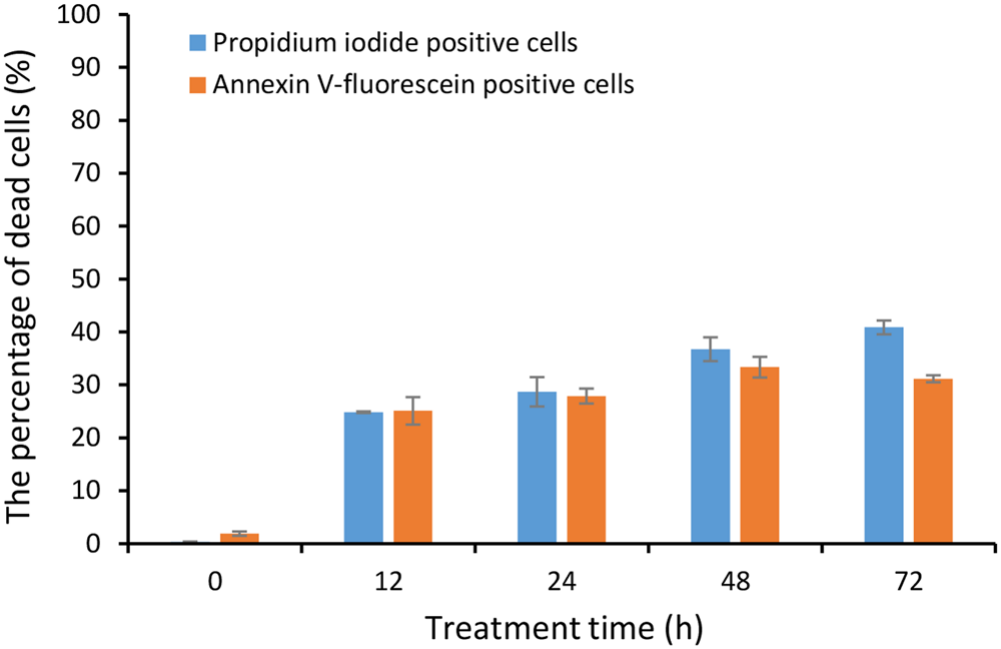
Flow cytometry analysis of cell membrane integrity. PG-treated *M. aeruginosa* cells (30 μg/mL) stained with propidium iodide (PI) and Annexin V-fluorescein.

To investigate the effect of PG on the cellular membrane of *M. aeruginosa*, propidium iodide (PI) and Annexin V-fluorescein (Annexin V-Fluos) fluorescence were analysed by flow cytometry. Figure 3 shows that the number of cells positively stained by PI continued to increase as the PG treatment time was prolonged. Approximately 25% of cells after 12 h and 41% of cells after 72 h exposure were positively stained by PI. The number of cells positively stained by Annexin V-Fluos also increased by approximately 25% after 12 h exposure and by more than 33% within 48 h.

### DNA fragmentation

**Figure 4:**
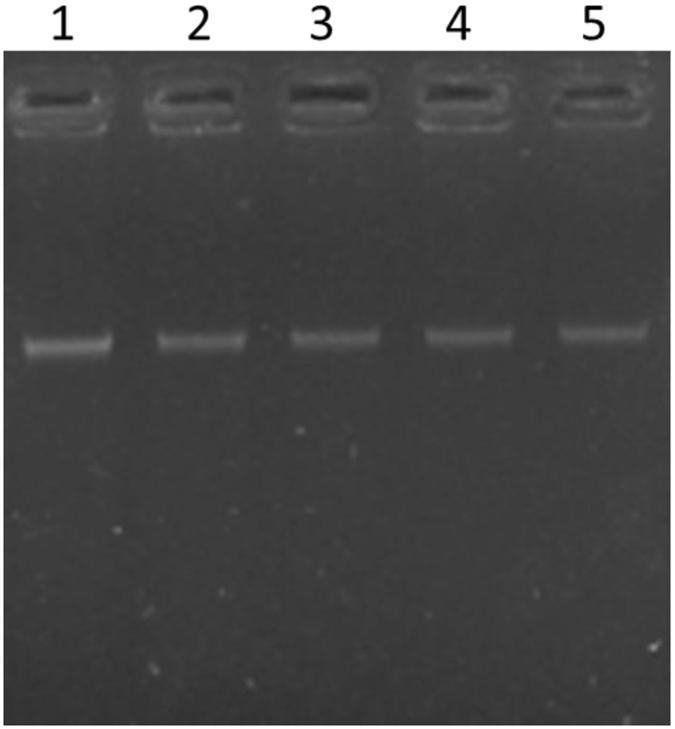
Agarose gel electrophoresis analysis of DNA. Total DNA was extracted from algal cells treated with DMSO (lane 1) or with 30 μg/mL PG for 12 h (lane 2), 24 h (lane 3), 48 h (lane 4) and 72 h (lane 5), and loaded onto 1% agarose gels.

Algal DNA was extracted from PG-treated cells. The DNA from dimethyl sulphoxide (DMSO)-treated cells was used as a control. We did not detect DNA laddering or smearing when the genomic DNA of PG-treated cells was electrophoresed (Fig. 4). This suggests that PG-treated *M. aeruginosa* undergoes cell death without the characteristic DNA degradation.

### Effect of PG on the ultrastructure of *M. aeruginosa*

**Figure 5:**
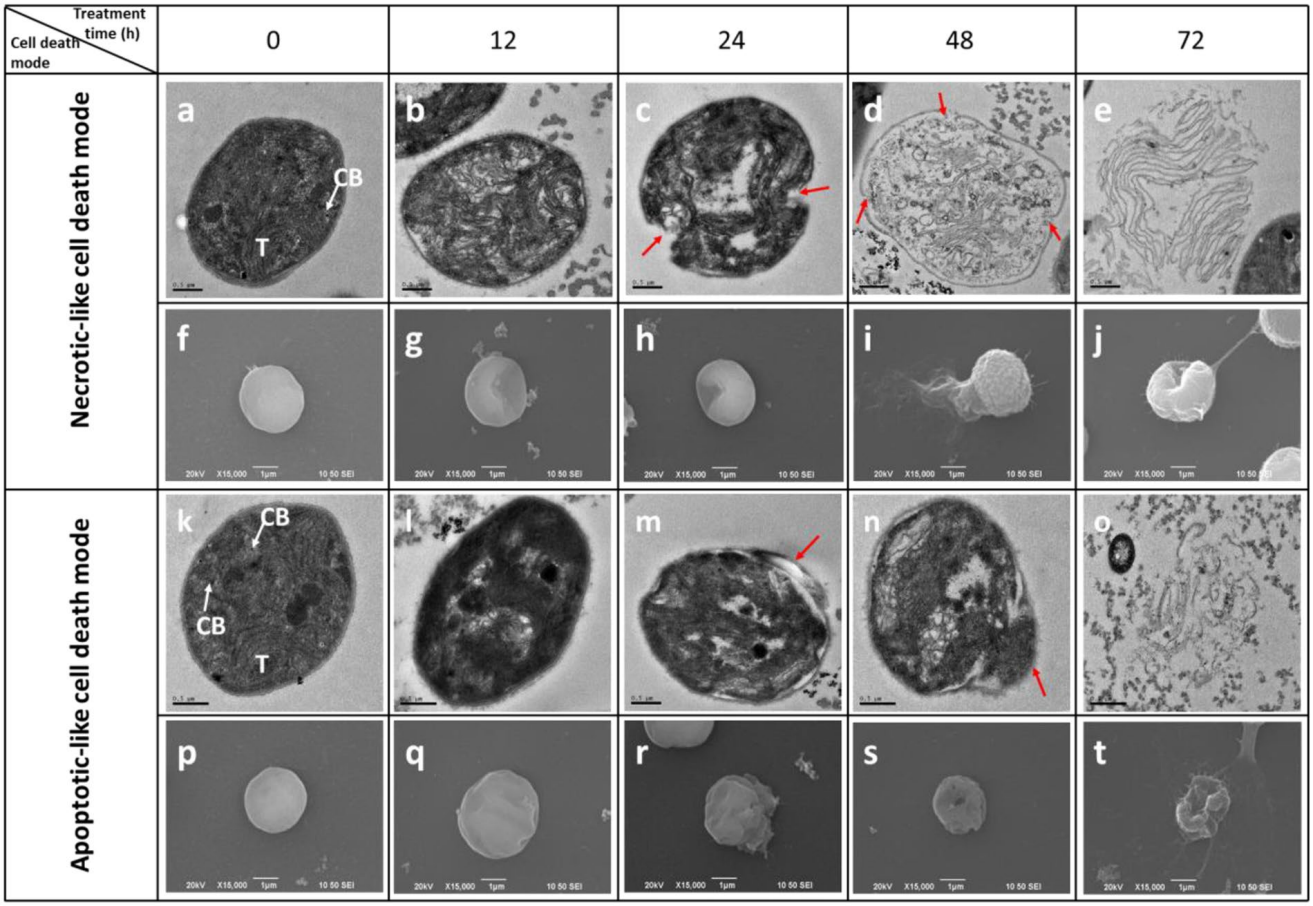
Two cell death modes in *M. aeruginosa* visualized by TEM and SEM. Damaged cells of *M. aeruginosa* treated with PG (30 µg/mL) for 12, 24, 48 and 72 h. (**a**,**f**,**k** and **p**): control cell; (**b**–**e** and **g**–**j**): necrotic-like cell death mode; (**l**–**o** and **q**–**t**): apoptotic-like cell death mode. (T: thylakoids; CB: carboxysome). Red arrows show where the cell membrane was broken. TEM images: bars = 0.5 µm; SEM images: bars = 1 µm.

To understand the cell death process of *M. aeruginosa*, the ultrastructure of algal cells was observed using transmission electron microscopy (TEM) and scanning electron microscopy (SEM). The control cells without PG treatment appeared healthy, with intact cellular characteristics, including cell membrane integrity, a homogeneous cytoplasm and a regularly arranged thylakoid (Fig. 5a,f,k,p). However, most cells under PG stress were dying or dead. A careful inspection of the dying cells at the ultrastructural level revealed the existence of two cell death modes in *M. aeruginosa* (Fig. 5).

One is a necrotic-like cell death mode. In the necrotic-like cell death mode, the cellular content was slightly reduced at an early stage, while the plasma membrane remained intact (Fig. 5b). At the later stages, cells lost membrane integrity and the cell wall ruptured, resulting in the leakage of cellular contents (Fig. 5c,d). Eventually, only disintegrated thylakoid membranes were left (Fig. 5e). The second mode is an apoptotic-like cell death mode. This mode was characterized by a slightly wrinkled appearance with a shrunken cytoplasm that retracted from the cell wall at the early and intermediate stages, while the cell wall remained intact and some inclusions, such as carboxysomes, were retained (Fig. 5l,m). At the late stage, the budding and formation of apoptotic bodies were observed (Fig. 5m,n), and the intact cell structure disappeared (Fig. 5o).

The observations by SEM corresponded with the results of TEM. The control cells were spherical with a smooth cell surface (Fig. 5f,p). In the necrotic-like cell death mode, the cell membrane and cell wall were ruptured, which led to the leakage of cellular contents. Finally, only empty cell envelopes were observed (Fig. 5g,h,i,j). In the apoptotic-like cell death mode, the cells gradually became wrinkled and the cell shape was irregular. With increased exposure time, cell blebbing could be observed. During apoptotic-like cell death, the cell wall and plasma membrane remained intact without obvious leakage of cellular contents (Fig. 5q,r,s,t).

### Effects of ROS levels and lipid peroxidation in *M. aeruginosa*

**Figure 6:**
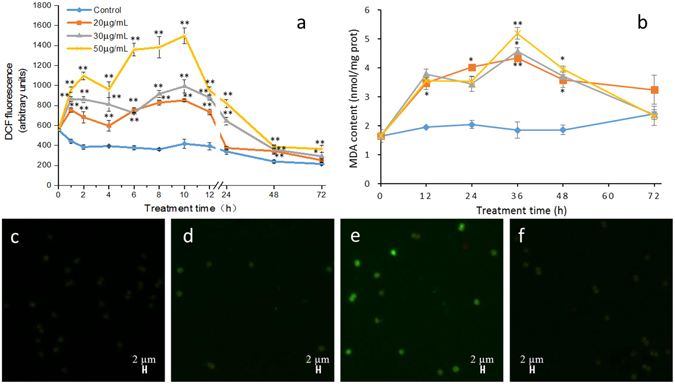
Effect of PG on ROS and MDA contents of M. aeruginosa. Algal cells treated with 20 µg/mL, 30 µg/mL or 50 µg/mL of PG were harvested for ROS levels (**a**) and MDA content (**b**) measurements. (**c**–**f**): fluorescence microscope images of intracellular ROS fluorescence intensity in cells treated with 30 µg/mL PG for 0, 2, 10 and 24 h. Values are means ± SD (n = 3). *(*p* < 0.05) and **(*p* < 0.01) represent significant differences compared to the control cells.

To evaluate the oxidative status caused by reactive oxygen species (ROS) in algal cells under PG stress, the ROS levels were assessed by dichlorofluorescin (DCF) fluorescence intensity. When algal cells were treated with different concentrations of PG (20, 30, and 50 µg/mL), the intracellular ROS levels increased significantly (*p* < 0.01) after 1 h of exposure and reached maximum levels after 10 h of exposure, at 2.0, 2.4, and 3.6 times those of the control cells, respectively (Fig. 6a). The ROS level of the treated cells decreased after 12 h of treatment but still remained higher than that of the control cells. Fluorescence microscopy images confirmed the changes in ROS. DCF fluorescence could not be detected in the 0 h treatment group (Fig. 6c), and there were few algal cells exhibiting green fluorescence after 2 h of treatment (Fig. 6d). After 10 h of exposure, almost all the cells showed bright DCF fluorescence (Fig. 6e). The intensity of DCF fluorescence became weak after 24 h of treatment (Fig. 6f).

Figure 6b shows that, similar to the ROS levels, the malondialdehyde (MDA) content in algal cells tended to increase after PG treatment. Compared to the control cells, the MDA content of treated cultures was significantly increased after exposure to different concentrations of PG for 12 h and reached maximum values at 36 h of exposure. The MDA contents of the cells treated with 20, 30, and 50 µg/mL PG for 36 h were 2.4 (*p* < 0.01), 2.5 (*p* < 0.05), and 2.8 (*p* < 0.01) times those of the control cells, respectively (Fig. 6b). The MDA content in the algal cells decreased with prolonged treatment time and reached the level of the control group after 72 h of treatment.

### Effects on the antioxidant system

**Figure 7:**
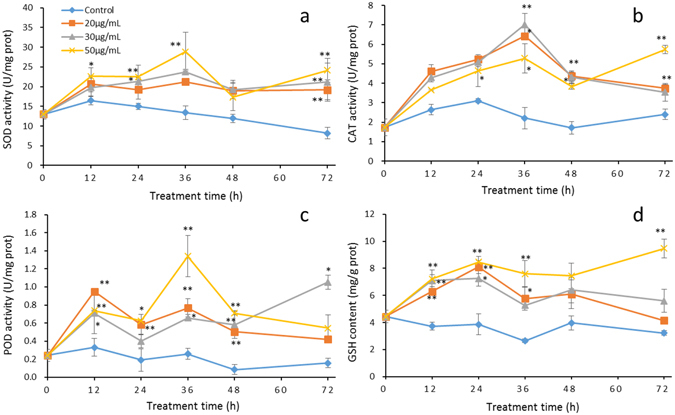
The response of the antioxidant system of *M. aeruginosa* to PG. Algal cells treated with 20 µg/mL, 30 µg/mL or 50 µg/mL of PG were harvested for activity analyses of SOD (**a**), CAT (**b**), POD (**c**), and GSH (**d**). Values are means ± SD (n = 3). *(*p* < 0.05) and **(*p* < 0.01) represent significant differences compared to the control cells.

To investigate the antioxidant defence response of algal cells induced by PG, the enzymatic antioxidants superoxide dismutase (SOD), catalase (CAT) and peroxidase (POD) and the non-enzymatic antioxidant glutathione (GSH) were measured. Figure 7 shows that the activity of these antioxidant defences in algae cultures treated with different concentrations of PG were markedly increased compared to the control cells. The activity of SOD (Fig. 7a) and CAT (Fig. 7b) increased with treatment time and reached a maximum within 36 h. After 36 h, SOD activity in PG-treated cells remained at high levels. Although the activities of CAT decreased slightly after PG exposure for 48 h, they were still higher than in the control cells (*p* < 0.01). The activity of POD (Fig. 7c) showed a very similar trend to SOD activity, increasing significantly (*p* < 0.01) after 12 h of exposure to different concentrations of PG and then remaining at relatively high levels. The contents of GSH increased significantly (*p* < 0.01) after exposure to PG for 12 h and reached a maximum within 24 h (*p* < 0.01). Although the contents of GSH in 20 and 30 µg/mL PG-treated cells decreased slightly after exposure for 36 h, the contents were still higher than in the control cells (Fig. 7d). These results suggest that algal cells activate antioxidant systems to protect the cells from oxidative damage under PG stress.

### Photosynthetic performance

**Figure 8:**
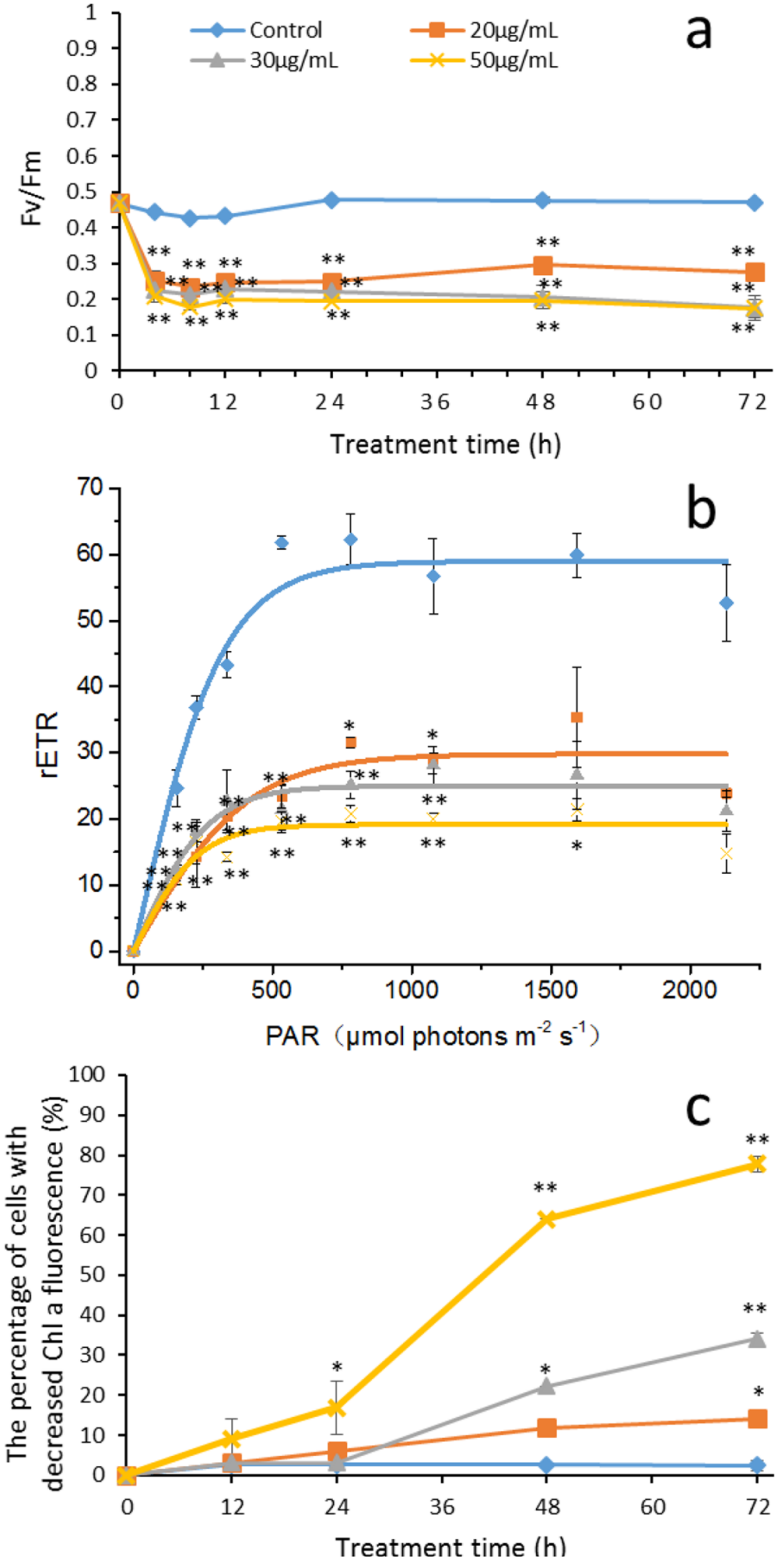
Effect of PG on (**a**) Fv/Fm, (**b**) rETR (4 h), and (**c**) Chl a fluorescence of *M. aeruginosa*. Values are means ± SD (n = 3). *(*p* < 0.05) and **(*p* < 0.01) represent significant differences compared to the control cells.

In an effort to investigate the photosynthetic status of algal cells under PG stress, the maximum quantum yield of photosystem (PS) II (Fv/Fm) and the relative electron transport rate (rETR) were measured. Fv/Fm represents photochemical efficiency of PS II, and rETR represents the photosynthetic rate. The Fv/Fm value of the cells exposed to 20, 30 and 50 µg/mL PG for 4 h was significantly decreased (*p* < 0.01), being 43%, 49% and 53% lower than the control value, respectively. With increased PG treatment times, the values of Fv/Fm remained stable and significantly lower than the control (*p* < 0.01), which implied that the photosynthetic capacity of those cells was inhibited (Fig. 8a). Similarly, the rETR value was also significantly decreased after treatment with PG for 4 h, implying that the photosynthetic efficiency of algal cells was inhibited by PG (Fig. 8b).

Figure 8c shows that the chlorophyll a (Chl a) autofluorescence of most cells became weak under the stress caused by 30 µg/mL and 50 µg/mL of PG. Within 48 h of exposure, 22% of cells in 30 µg/mL PG-treated culture showed weak Chl a autofluorescence, and this percentage increased to 64% in the 50 µg/mL PG-treated culture. As the treatment time was prolonged, the percentage continued to increase, to 34% in the 30 µg/mL PG-treated culture and 78% in the 50 µg/mL PG-treated culture. These results indicated that PG could damage Chl a.

### Effects on protein content

**Figure 9:**
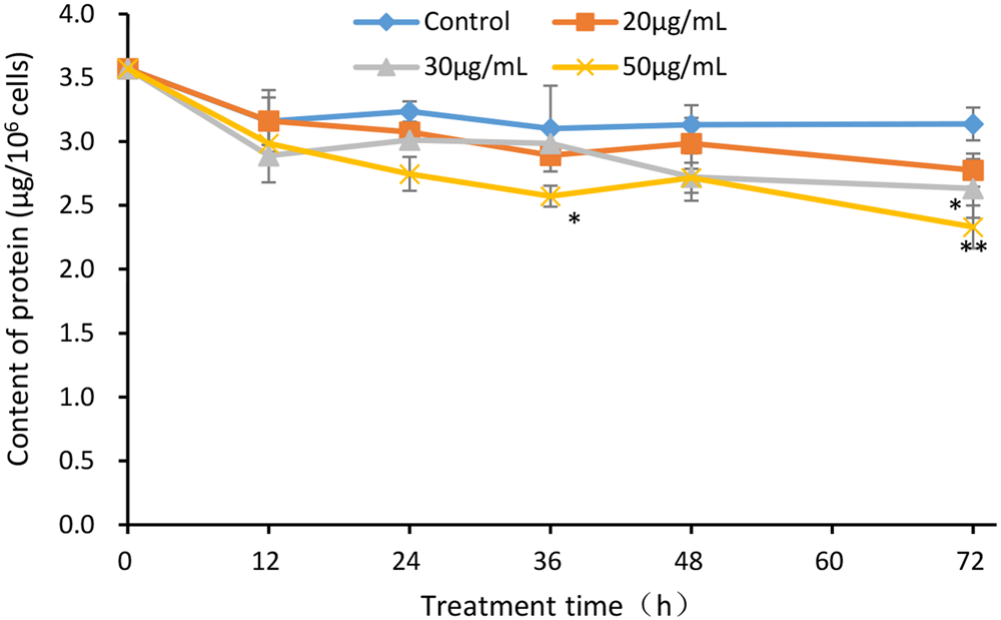
Effect of PG on protein content of *M. aeruginosa*. Values are means ± SD (n = 3). *(*p* < 0.05) and **(*p* < 0.01) represent significant differences compared to the control cells.

Figure 9 shows that the protein content of PG-treated cells declined gradually. The control sample contained ~3.2 μg of protein per 10^6^ cells. Living cells treated with 20 μg/mL, 30 μg/mL or 50 μg/mL PG for 72 h contained an average of 2.8 μg, 2.6 μg and 2.3 μg of protein per 10^6^ cells, respectively. Compared with the control culture, the protein contents were decreased by 12%, 16% (*p* < 0.05), and 26% (*p* < 0.01) in the 20, 30, and 50 μg/mL treatment cultures, respectively, after 72 h of exposure. These data indicated that PG treatment could affect protein synthesis and lead to protein degradation in *M. aeruginosa*.

### Proteomic analysis of algal cells under PG stress

To examine the cellular responses and molecular mechanisms associated with ROS production and cell-fate decisions of *M. aeruginosa* under PG stress, we harvested algal cells after treatment with 30 μg/mL PG at 12 h, 24 h, 48 h and 72 h for a tandem mass tag (TMT)-based proteomics analysis. Using TMT labelling and HPLC fractionation followed by high-resolution tandem mass spectrometry (LC-MS/MS) analysis, a quantitative global proteome analysis was performed. In total, 1,943 protein groups were identified from *M. aeruginosa*, from which 1,513 proteins were quantified. When setting the quantification ratio of >1.3 as the up-regulated threshold and <0.77 as the down-regulated threshold (*p* < 0.05), many differentially expressed proteins were obtained in each PG-treated group. As the treatment time increased, the number of differentially expressed proteins increased as well (Table 1).

**Table 1:** Summary of differentially quantified proteins (>1.3, or <0.77)

Sample ID	Up-regulated	Down-regulated
S2/S1	22	29
S3/S1	45	25
S4/S1	97	65
S5/S1	109	91

Control sample (S1); sample treated with PG for 12 h (S2), 24 h (S3), 48 h (S4), 72 h (S5).

**Figure 10:**
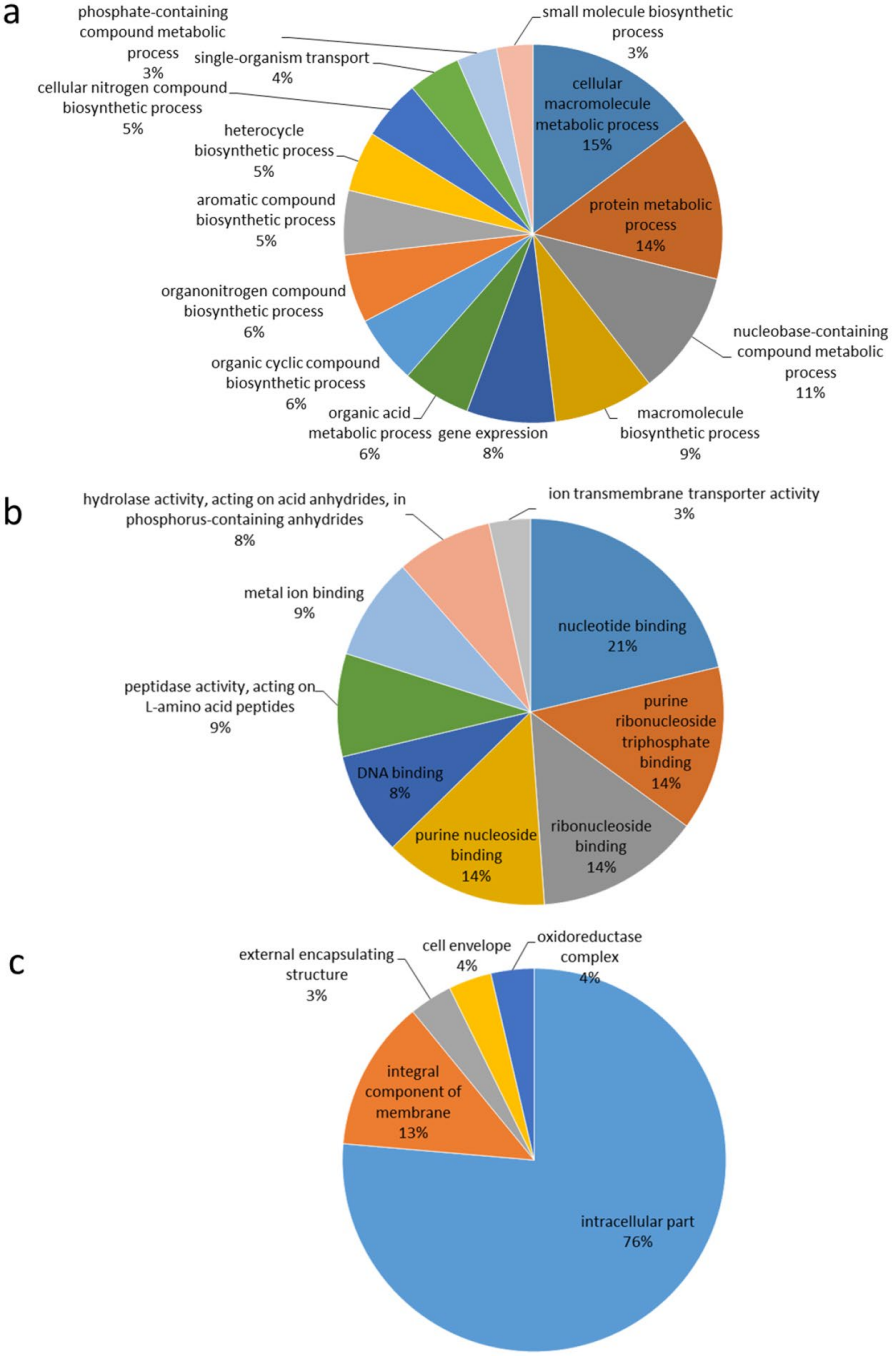
Gene ontology-based functional characterization of the differentially expressed proteins in *M. aeruginosa* under PG stress. Distribution of the differentially expressed proteins in terms of biological processes (**a**), molecular function (**b**), cellular components (**c**).

To investigate the cellular functions of the differentially expressed proteins in *M. aeruginosa*, we classified these proteins into different groups according to biological processes, molecular function, and cellular components. The functional characterization of these proteins was further assigned according to gene ontology (GO) term annotation (Fig. 10). Among these proteins, 15% are cellular macromolecule metabolic process proteins, 14% are related to protein metabolic processes and 11% are involved in nucleobase-containing compound metabolic processes (Fig. 10a). By grouping differentially expressed proteins by their molecular function, we observed that, 21% have nucleotide binding activitiy, 14% have purine ribonucleoside triphosphate binding activity, 14% have ribonucleoside binding activity and 14% show purine nucleoside binding activity, suggesting that these proteins are involved in DNA repair, transcription and translation. In addition, 9% of the proteins were associated with peptidase activity, indicating that these proteins are responsible for protein degradation (Fig. 10b). Most of the identified proteins are localized to subcellular components that are related to their functions in the cell. The GO analysis showed that 76% of the proteins are intracellular, 13% are integral membrane components, 4% are located in cell envelopes, 4% are associated with oxidoreductase complexes, and 3% are part of the external encapsulating structure (Fig. 10c).

**Figure 11:**
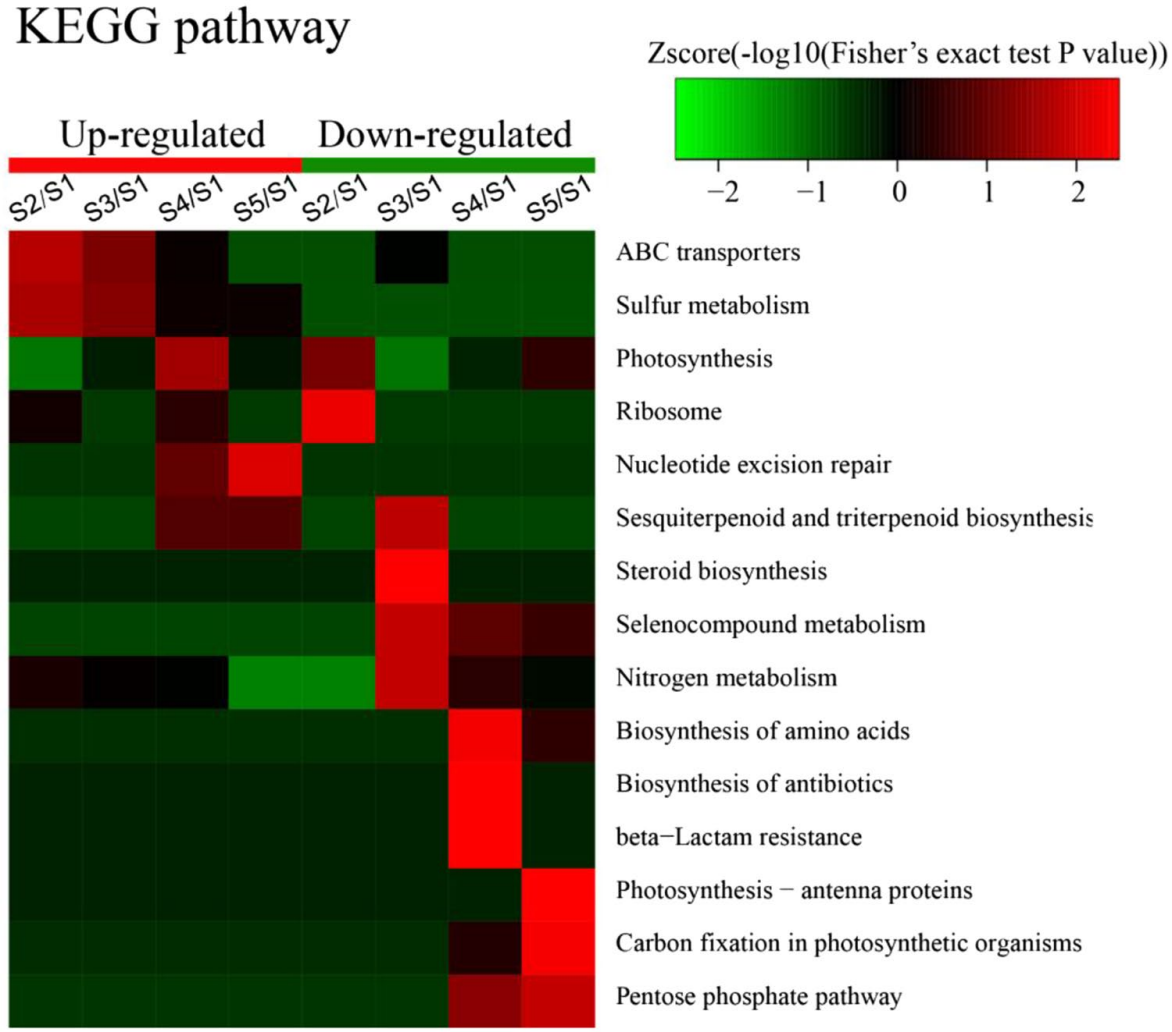
Heat maps obtained from GO enrichment-based cluster analysis of KEGG pathways. Control sample (S1); sample treated with PG for 12 h (S2), 24 h (S3), 48 h (S4), 72 h (S5).

To gain insight into the metabolic response to PG in *M. aeruginosa*, we conducted a KEGG pathway analysis of the differentially expressed proteins. The results showed that all differentially expressed proteins are involved in 15 metabolic pathways (Fig. 11).

The up-regulated proteins were enriched in the categories of ABC transporters, sulphur metabolism and nucleotide excision repair. The proteins in the categories of ABC transporters and sulphur metabolism exhibited up-regulated expression in algal cells after exposure to PG for 12 h and 24 h (Fig. 11). The proteins associated with nucleotide excision repair were up-regulated after exposure to PG for 48 h and 72 h (Fig. 11).

The down-regulated proteins were enriched in the categories of ribosome, biosynthesis and photosynthesis. At the early stage of PG treatment (12 h, Fig. 11-S2), the proteins associated with the ribosome category were down-regulated, suggesting the inhibition of translation under PG stress. The proteins related to nitrogen metabolism and the biosynthesis of macromolecules, including sesquiterpenoids, triterpenoids, steroids, amino acids and antibiotics, were down-regulated at the intermediate stage (24 h and 48 h, Fig. 11-S3, S4), indicating the inhibition of metabolic processes. Finally, all aspects of photosynthesis, including photosynthesis antenna proteins, and proteins involved in carbon fixation and the pentose phosphate pathway, were down-regulated after 72 h of PG treatment (Fig. 11-S5), indicating the dysfunctioning of the photosynthetic system.

## Discussion

In recent years, PGs have attracted much attention for their potential to control harmful algal blooms. Zhang *et al*. previously isolated and identified a *Hahella* strain with high algicidal activity against *P. globosa*, which is implemented through the production of PG^27^. PG was also reported to have high algicidal activity against *M. aeruginosa* ^26^, but further knowledge about its algicidal mechanism is needed. In this study, we explored the algicidal effect and mechanism of PG from *Hahella* sp. KA22 against *M. aeruginosa*, and found that *M. aeruginosa* undergoes programmed cell death (PCD) under PG stress.

Previously, PCD was thought to only exist in eukaryotic organisms. However, an increasing body of evidence suggests that PCD also occurs in prokaryotic organisms including *Escherichia coli*, *Anabaena* and *Trichodesmium* ^30–33^. Zheng *et al*. reported that cyanobacteria initiated PCD after being subjected to darkness for five days. TEM observation showed four cell death modes: apoptotic-like, autophagic-like, necrotic-like and autolytic-like^34^. In *M. aeruginosa* under PG stress, we observed two cell death modes using TEM and SEM. One is necrotic-like cell death. In this cell death mode, the disruption of cell membranes and the release of cellular contents were observed after exposure to PG. The other mode is apoptotic-like cell death. The budding and formation of apoptotic bodies were observed after algal cells were treated with PG. These results agree with the report by Zheng *et al*.^34^ and indicate that PG initiates PCD in *M. aeruginosa*. Hu *et al*. also found that cinnamaldehyde-induced PCD occurs in *M. aeruginosa* ^35^.

During PCD, the cell membrane usually shows characteristic changes, such as phosphatidylserine externalization or membrane permeabilization. Some studies have reported that algicidal compounds can alter membrane permeability and damage the cell membrane, eventually leading to the leakage of cell contents^36–38^. Li *et al*. reported that algicidal extracts from *Mangrovimonas yunxiaonensis* strain LY01 can change the cell membrane permeability of *Alexandrium tamarense* as well as disrupt the cell membrane^39^. To learn more about the alteration of the cell membrane during PG-induced cell death in *M. aeruginosa*, we carried out PI and Annexin V-Fluos assays. The PI assay, which demonstrates membrane permeabilization, is commonly used as an indicator of necrotic cells or late-stage apoptotic that have lost cell membrane integrity, and the Annexin V-Fluos assay, which registers externalized phosphatidylserine (PS) at the plasma membrane, is used as an indicator of early apoptosis. Our results showed that the number of algal cells with PI positive staining or Annexin V-Fluos positive staining rose with the treatment time of PG. These results indicated that PG causes the externalization of phosphatidylserine and the loss of membrane integrity, which suggest that the cell death modes of *M. aeruginosa* under PG stress are apoptotic-like and necrotic-like.

ROS, including hydroxyl radicals and hydrogen peroxide, are produced as by-products of various metabolic pathways and physiological processes such as photosynthesis and respiration. Sometimes, cells generate ROS as signalling molecules to control different biological processes including pathogen defence and cell cycle, but excess ROS can cause oxidative damage to lipids, proteins, and DNA and eventually lead to cell death^40^. ROS are usually overproduced when algal cells grow under adverse circumstances such as high temperature, high light, and in the presence of allelochemicals^41–43^. Zhang *et al*. reported that *A. tamarense* generates excessive ROS when treated with the supernatant of *Vibrio* sp. DHQ25 cultures, which initiates lipid peroxidation and results in cell death^44^. Both Li *et al*.^45^ and Lei *et al*.^46^ reported that an algicidal component triggered excessive ROS production that damaged the cell membrane and photosystem of *A. tamarense* and ultimately caused cell death. Guo *et al*. report that two diketopiperazines produced by *Chryseobacterium* sp. increased intracellular ROS levels in *M. aeruginosa* and caused irreversible oxidative damage to algal cells^47^. Our results indicate that PG triggers ROS overproduction in *M. aeruginosa* at the early stage and that the overproduction of ROS increases the content of MDA, which is usually used as an indicator of lipid peroxidation and a biomarker for oxidative stress^48^. This indicates that the excess intracellular ROS initiated lipid peroxidation and caused oxidative damage to cellular membranes, such as plasma and thylakoid membranes. In addition, the overproduction of ROS stimulated the activity of the antioxidant system to counteract the oxidative stress. To scavenge ROS, algae cells have a defence system involving enzymatic antioxidants, such as SOD, CAT and POD, and non-enzymatic antioxidants, such as GSH. Many researchers have reported that allelochemicals including nanaomycin A methyl ester^49^, salcolin^50^, and deinoxanthin^51^ can induce cells to produce excessive ROS, which in turn activates the antioxidant system to scavenge ROS and protect cells from oxidative damage. Our results showed that the activity of SOD, CAT, POD and GSH were enhanced under PG stress. Therefore, ROS levels and MDA content decreased under prolonged exposure to PG. Although ROS levels under PG treatment decreased markedly after 12 h of exposure and could be partially eliminated by the increasing activity of the antioxidant system, these levels were still much higher than those of the control cells. In accordance with the high level of ROS, MDA contents were also significantly increased under PG treatment. We suggest that excess ROS resulted in oxidative damage in algal cells and probably acted as a signal to elicit PCD in *M. aeruginosa*.

A number of articles report that the photosynthetic apparatus is sensitive to adverse environmental factors such as allelochemicals^43^, high light^52^ and UV-B radiation^53^. Chl a is the main photosynthetic pigment in cyanobacteria and plays a crucial role in light harvesting and energy conversion. Most of the algal cells in the PG-treated groups showed decreased Chl a content. Meanwhile, photochemical efficiency and relative electron transport rate of algal cells were also seriously inhibited. These results indicate that PG can cause the dysfunctioning of photosynthesis by degrading Chl a and disrupting the photosynthetic system. Both the primary photochemical reaction and electron transport are accompanied by the movement of electron carriers and/or related enzymes within the thylakoid membrane. Therefore, photosynthesis is mainly affected by the structure and fluidity of the thylakoid membrane^54^. The significant inhibitions of photochemical efficiency and relative electron transport rate in PG-treated cells indicate that PG can cause the dysfunction of photosynthetic systems by disrupting the thylakoid membrane. We suggest that the excess ROS is responsible for the degradation of Chl a and the damage to the thylakoid membrane. Furthermore, our proteomics analysis indicates that PG influences every aspect of photosynthesis, including the synthesis of photosynthesis antenna proteins, carbon fixation and the pentose phosphate pathway.

The content of protein can indirectly reflect the growth state of algal cells. The protein contents in *M. aeruginosa* cells treated with PG were markedly decreased and most cells were dead or dying. The GO analysis indicated that the differentially expressed proteins are involved in a wide range of biological processes and have different molecular functions. The enrichment-based cluster analysis of KEGG pathways suggested that PG not only induced ROS overproduction to cause oxidative damage in algal cells, but also altered various metabolic pathways and biological processes. The up-regulated proteins were enriched in the categories of ABC transporters, sulphur metabolism and nucleotide excision repair related pathways. From this result, we infer that the transmembrane transport of PG is probably carried out by ABC transporters and PG stimulates sulphur metabolism in *M. aeruginosa*. Typically, the increase in nucleotide excision activity and DNA degradation accompany cell death. However, we did not detect DNA degradation characteristic of cell death. Based on the proteomics analysis, we conclude that the up-regulated expression of proteins associated with nucleotide excision repair blocked DNA degradation. The down-regulated proteins were enriched in the categories of ribosome, nitrogen metabolism, amino acid biosynthesis and photosynthesis. Therefore, we suggest that PG probably targets the ribosome, interferes with nitrogen metabolism, down-regulates the biosynthesis of amino acids, and eventually leads to a decrease in the protein content of algal cells. In the meantime, PG treatment resulted in the dysfunctioning of the photosynthetic system, likely through the degradation of photosynthesis antenna proteins and those proteins involved in carbon fixation and the pentose phosphate pathway. All of these pathways could be directly related to the stress response of *M. aeruginosa* to PG.

**Figure 12:**
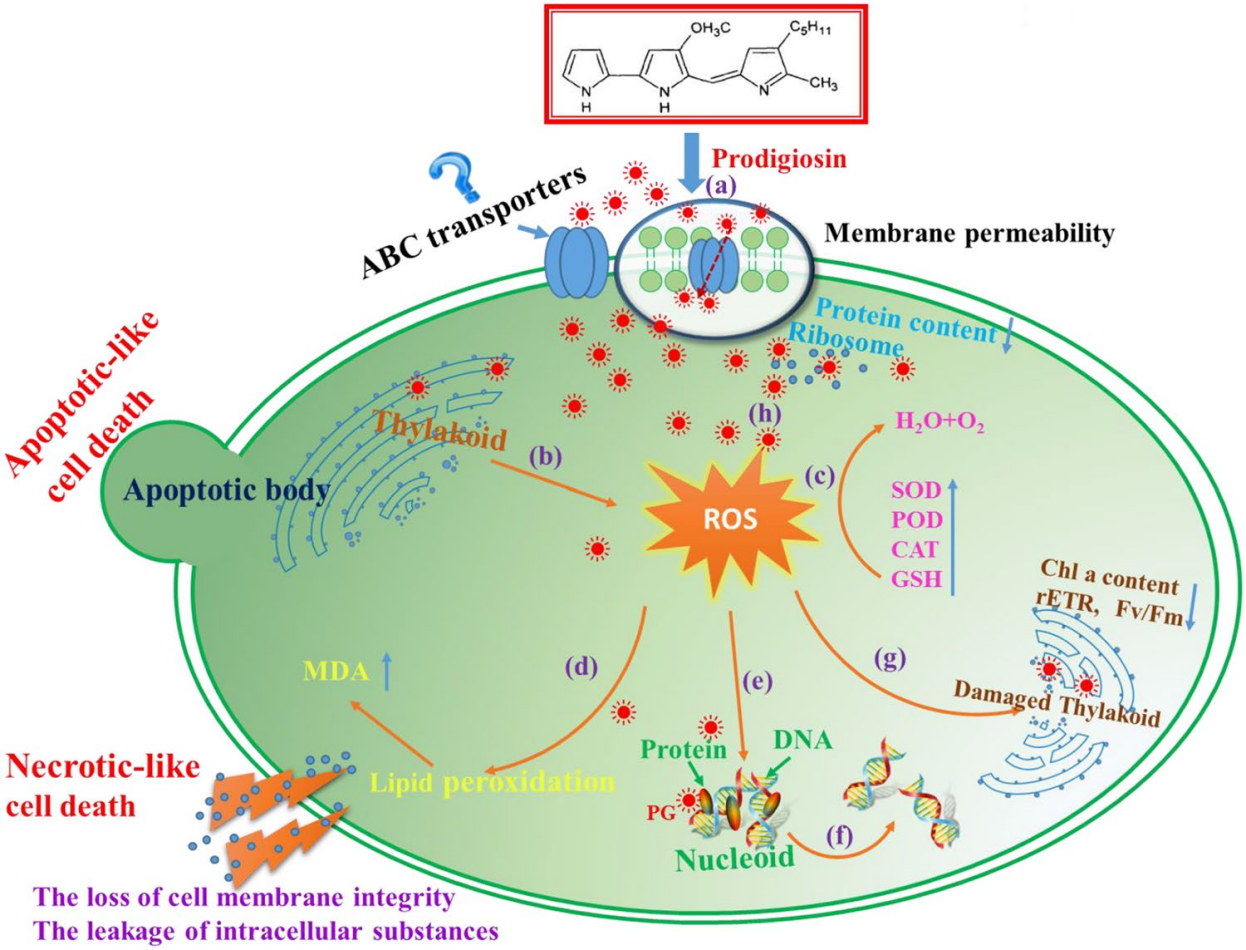
A schematic diagram depicting the mechanism of algal cell death under PG stress. (**a**) PG may be transferred across the cell membrane by ABC transporters; (**b**) PG likely stimulates photosynthesis to generate excess ROS; (**c**) the antioxidant system, including GSH, SOD, POD and CAT, are activated to scavenge ROS; (**d**) the excess ROS could lead to lipid peroxidation, and the cellular membrane permeability and integrity are damaged, which likely results in necrotic-like cell death; (**e**) PG may bind to nucleotides, especially purine (ribo)nucleosides, and the excess ROS could cause DNA damage; (**f**) the damaged DNA may be partially repaired by the nucleotide excision repair system; (**g**) the excess ROS could disrupt the thylakoid membrane and inhibit photosynthesis as the treatment time increases; (**h**) PG may target the ribosomes, which could interfere with protein synthesis.

Based on our studies, we suggest a possible mechanism of *M. aeruginosa* cell death under PG stress (Fig. 12). PG is probably transferred across the plasma membrane by specific ABC transporters and then stimulates the overproduction of ROS in algal cells. Although excess ROS activates the antioxidant system to protect against oxidative damage, the content of ROS remains at a relatively high level. The surplus ROS initiate lipid peroxidation and cause oxidative damage to membrane systems, including plasma membranes and thylakoid membranes. The damage to the thylakoid membranes leads to the dysfunctioning of the photosynthetic system. Furthermore, ROS probably act as signalling molecules to trigger apoptotic-like and necrotic-like cell death in *M. aeruginosa*. PG not only initiates the overproduction of ROS, but also influences various metabolic processes including sulphur metabolism and nitrogen metabolism, binds to nucleotides, and potentially targets ribosomes to disrupt peptide synthesis. Although DNA fragmentation has been described as a characteristic of PCD in cyanobacteria^32, 34^, we did not observe this during PG-induced PCD of *M. aeruginosa*. It is likely that PG activates the DNA excision repair system to repair damaged DNA.

In conclusion, our results indicate that PG is an effective algicidal compound against *M. aeruginosa*. It induced ROS production, destroyed membrane systems, inhibited photosynthesis, decreased Chl a and protein contents, altered the antioxidant system, affected whole protein expression modes and eventually initiated apoptotic-like and necrotic-like cell death. Furthermore, PG is degradable under natural light^27^. This poses an advantage for its potential use to control *M. aeruginosa* blooms because it would be degraded naturally in the environment.

## Materials and Methods

### *Microcystis aeruginosa* culture

*Microcystis aeruginosa* TAIHU98 (Collection No. FACHB-1752), was obtained from the Freshwater Algae Collection at the Institution of Hydrobiology (FACHB-collection), Chinese Academy of Sciences. *M. aeruginosa* was maintained as a unialgal axenic culture in sterile BG11 medium^55^ at 25 ± 1 °C with illumination at 50 μmol photons m^−2^ s^−1^ under a 12:12 h light-dark cycle. Exponential phase algal cultures were used for the following experiments.

### Isolation and purification of prodigiosin

Prodigiosin (PG) was extracted from *Hahella* sp. KA22, which had been isolated from the surface sediment of the Yunxiao Mangrove National Nature Reserve by Zhang *et al*.^27^. The strain KA22 was cultured in Zobell 2216E broth (peptone 5 g, yeast extract 1 g, ferric phosphate 0.1 g, in 1 L natural sea water, pH 7.6–7.8) at 28 °C with rotation at 150 rpm for 24 h. PG was extracted and purified according to the method reported by Zhang et al.^27^. The purified PG was dissolved in dimethyl sulphoxide (DMSO).

### Algicidal activity of PG

The exponentially growing cells of *M. aeruginosa* were treated with various concentrations of PG (5, 10, 20, 30 and 50 μg/mL) and the control was treated with the same volume of DMSO. The growth of *M. aeruginosa* was monitored using a microplate reader (SpectraMax M2, Molecular Devices, Sunnyvale, USA) by measuring chlorophyll fluorescence (λex = 440 nm, λem = 680 nm). The algicidal activity was calculated according to the formula below.

$${\rm{Algicidal\; activity}}( \% )=({\rm{Fc}}-{\rm{Ft}})/{\rm{Fc}}\times 100 \% $$

Where Fc and Ft are the fluorescence intensity of the control and treatments, respectively^56^. LD_50_ was calculated using SPSS after treatment with 5, 10, 20, 30 and 50 μg/mL PG for 72 h. The algicidal effect of PG against *M. aeruginosa* was observed microscopically (Olympus BX41, Tokyo, Japan). All experiments were repeated in triplicate.

### Nucleoid analysis

After being treated with PG, *M. aeruginosa* cells were harvested using centrifugation at 5,000 rpm for 5 min. The cell pellet was resuspended and fixed for 10 min at room temperature in 4% paraformaldehyde (v/v in 0.1 M phosphate buffer saline (PBS), pH 7.4). After being washed twice with PBS, the fixed cells were stained with 5 µg/mL DAPI for 5 min in the dark at room temperature. The fluorescence was evaluated using a fluorescence microscope^57^.

### Flow cytometer analysis

*M. aeruginosa* cells were harvested by centrifugation at 5,000 rpm for 5 min and then washed twice with PBS (0.1 M, pH 7.4). The cell pellet was resuspended in 100 µL propidium iodide (PI) and Annexin V-Fluos labelling solution containing Annexin V-fluorescein (Annexin V-Fluos Staining Kit, Roche, USA) and incubated for 15 min in the dark at 20 °C. Fluorescence was analyzed on a BD LSRFortessa flow cytometer (BD, USA) using 488 nm excitation and a 525 nm bandpass filter for Annexin V-fluorescein detection and a 630 nm bandpass filter for PI^58, 59^. For each sample about 30,000 cells were analyzed.

### DNA fragmentation

*M. aeruginosa* cells were harvested by centrifugation at 5,000 rpm for 5 min. Cell pellets were washed twice with buffer A (50 mM Tris-HCl pH 8.0, 5 mM EDTA, 50 mM NaCl) and resuspended in buffer B (50 mM Tris-HCl pH 8.0, 50 mM EDTA) containing 15 mg/mL lysozyme (Sangon Biotech Ltd, Shanghai, China). The cell suspension was incubated at 37 °C for 1 h and then the same volume of buffer C (Tris 30 mM, EDTA 10 mM, sarkosyl 2% (w/v)) containing 15 µg/mL RNase H (TakaRa Bio Inc, Dalian, China) was added. After incubation at 37 °C for 45 min, DNA was extracted twice with phenol-chloroform and separated on a 1% agarose gel.

### Transmission and scanning electron microscopy

Algal cells were harvested by centrifugation at 3,500 rpm for 10 min after the cultures were treated with PG (30 μg/mL) for 12, 24, 48 and 72 h. The collected cells were fixed overnight at 4 °C in PBS (0.1 M, pH 7.4) containing 2.5% glutaraldehyde (v/v) and then washed three times with PBS. The cells were post-fixed in 1% OsO_4_ (v/v in 0.1 M PBS) for 2 h and then dehydrated in a graded ethanol series (30, 50, 70, 90 and 100%). Samples were embedded in LR white. Ultrathin sections (60–80 nm) were stained with 3% acetic acid uranium-citric acid and observed by transmission electron microscopy (JEM-2100HC, JEOL Co., Japan).

SEM samples were collected and fixed as described above. The fixed cells were attached onto a cover slip and dehydrated in a graded ethanol series (30, 50, 70, 90, 95 and 100%) and then critical-point-dried. The dry cells were sputter-coated with gold and then viewed using a scanning electron microscope (JSM-6390, JEOL Co., Japan)^60, 61^.

### Determination of ROS levels, MDA content and antioxidative enzyme activities

Intracellular reactive oxygen species (ROS) content was determined using a fluorogenic probe, 2′, 7′-dichlorofluorescin diacetate (DCFH-DA, Nanjing Jiancheng Bioengineering Institute, China), based on the method described by Zhang *et al*.^62^, with slight modifications. The algae cultures were treated with different concentrations of PG (20, 30 and 50 μg/mL) and samples were collected after 0, 1, 2, 4, 6, 8, 10, 12, 24, 48 and 72 h exposure, while algae cultures mixed with the same volume of DMSO served as the control. Cells were centrifuged at 4,000 g for 10 min. 0.5 mL 10 μM DCFH-DA dissolved in BG11 medium was added in an Eppendorf tube (1.5 mL) to resuspend the pellet and was incubated for 1 h in the dark at 37 °C. The cells were washed twice with sterile BG11 medium and resuspended in sterile BG11 medium (0.5 mL). The DCF fluorescence was monitored using a microplate reader (λex = 488 nm, λem = 525 nm) and a fluorescence microscope.

Malondialdehyde(MDA) was determined by the Microscale MDA assay kit (Nanjing Jiancheng Bioengineering Institute, China). Algal cells were collected using centrifugation at 6,000 rpm for 10 min, and then washed twice with PBS (0.1 M, pH 7.4). Cells were resuspended in PBS (1.5 mL) and sonicated on ice using the Ultrasonic Cell Disruption System (NingBo Scientiz Biotechnological Co., Ltd, China) (80 W, 5 s: 5 s, 100 times). The remaining debris was removed by centrifugation at 15,000 rpm at 4 °C for 10 min. The supernatant was taken for the MDA measurement according to the MDA assay kit’s Operation Manual^63^. The content of protein was measured using a total protein quantitative assay kit (Nanjing Jiancheng Bioengineering Institute, China) and bovine serum albumin as the standard. The enzymatic activities of superoxide dismutase (SOD), peroxidase (POD), catalase (CAT) and the non-enzymatic antioxidant content of glutathione (GSH) was determined by Diagnostic Reagent Kits (Nanjing Jiancheng Bioengineering Institute, China) according to the kit’s Operation Manual. The activity of SOD was measured using the xanthine oxidase method based on the production of O_2_^−^ **•**. O_2_^−^ **•** oxidize hydroxylamine and produce a red substance after 20 min of reaction time at 37 °C. This was determined by absorbance at 450 nm. The activities of POD and CAT were measured by analyzing the decomposition rate of H_2_O_2_ at 420 nm and 405 nm. The content of GSH was detected based on the principle that GSH reacts with 5, 5′-dithiobis (2-nitrobenzoic acid) (DTNB) to produce a yellow substance which was determined by absorbance at 420 nm.

### Chlorophyll fluorescence measurements

To assay the photosynthetic response of the algal cells exposed to different concentrations of PG (20, 30 and 50 μg/mL), the maximum quantum yield of photosystem (PS) II (Fv/Fm) and the relative electron transport rate (rETR) were investigated using fast chlorophyll fluorescence on a pulse-amplitude modulation fluorometer (XE-PAM, Walz, Effeltrich, Germany). Fv/Fm was measured after the algal cells were incubated in darkness for 15 min^64, 65^.

### Total protein extraction and LC-MS/MS analysis

The sample was first ground with liquid nitrogen, then the cell powder was transferred to a 5 mL centrifuge tube and was sonicated three times on ice using a high intensity ultrasonic processor in lysis buffer (8 M urea, 1% Triton-100, 65 mM DTT and 0.1% Protease Inhibitor Cocktail). The remaining debris was removed by centrifugation at 16,000 rpm at 4 °C for 10 min. The protein was precipitated with cold 15% trichloroacetic acid for 2 h at −20 °C. After centrifugation at 4 °C for 10 min, the supernatant was discarded. The remaining precipitate was washed with cold acetone three times. The remaining protein was dissolved in buffer (8 M urea, 100 mM Tetraethylammonium bromide, pH 8.0) and the protein concentration was determined with the 2-D Quant kit according to the manufacturer’s instructions.

The proteins were digested with trypsin at a 1:50 trypsin-to-protein mass ratio for the first digestion overnight and at a 1:100 trypsin-to-protein mass ratio for a second 4 h-digestion. After Tandem Mass Tag (TMT) labeling, equal amounts of labeled peptides from each group were mixed and dissolved in 0.1% formic acid (FA) and directly loaded onto a reversed-phase pre-column (Acclaim PepMap 100, Thermo Scientific, Waltham, USA). Peptide separation was performed using a reversed-phase analytical column (Acclaim PepMap RSLC, Thermo Scientific). The resulting peptides were subjected to Nanospray ionization resource source followed by tandem mass spectrometry (MS/MS) in a mass spectrometer (Q Exactive^TM^ Plus LCMS, Thermo Scientific) coupled online to the UPLC. Intact peptides were detected in the Orbitrap at a resolution of 70,000. Peptides were selected for MS/MS using normalized collision energy setting as 30; ion fragments were detected in the Orbitrap at a resolution of 17,500.

### Proteomic data treatment and bioinformatic analyses

The resulting MS/MS data were processed using the Mascot search engine (v.2.3.0). Tandem mass spectra were searched against the *M. aeruginosa* database (5,571 sequences). Trypsin/P was specified as the cleavage enzyme allowing up to 2 missing cleavages. Mass error was set to 10 ppm for precursor ions and 0.02 Da for fragment ions. Carbamidomethyl on Cys, TMT-6plex (N-term) and TMT-6plex (K) were specified as fixed modification and oxidation on Met was specified as variable modifications. False discovery rate was adjusted to <1% and peptide ion score was set >20. All identified and quantified proteins were classified by gene ontology (GO) annotation into three categories: biological process, cellular compartment and molecular function using UniProt-GOA database (http://www.ebi.ac.uk/GOA/). For each category, a two-tailed Fisher’s exact test was employed to test the enrichment of the differentially expressed proteins against all identified proteins. Correction for multiple hypothesis testing was carried out using standard false discovery rate control methods. The GO with a corrected *p* < 0.05 is considered significant. The KEGG database was used to identify enriched pathways. The pathway with a corrected *p* < 0.05 was considered significant.

### Statistical analyses

Statistical differences of all data in this study were tested with analysis of variance (ANOVA) followed by the least significant difference test, with *p* < 0.01 and *p* < 0.05 considered significant (SPSS 19.0 for windows).
